# An extended 3D whole-heart myocardial first-pass perfusion sequence: alternate-cycle views with isotropic and high-resolution imaging

**DOI:** 10.1186/1532-429X-18-S1-Q60

**Published:** 2016-01-27

**Authors:** Merlin J Fair, Peter Gatehouse, Edward V DiBella, Liyong Chen, Ricardo Wage, David Firmin

**Affiliations:** 1 grid.7445.20000000121138111NHLI, Imperial College London, London, United Kingdom; 2 grid.439338.6CMR Unit, Royal Brompton Hospital, London, United Kingdom; 3 grid.223827.e0000000121930096UCAIR, University of Utah, Salt Lake City, UT USA; 4 grid.47840.3f0000000121817878UC Berkeley, Berkeley, CA USA; 5 grid.422032.5Advanced MRI Technologies, Sebastopol, CA USA

## Background

Simultaneously optimising parameters such as LV coverage, image resolution and contrast sensitivity is difficult in first-pass perfusion (FPP). 3D FPP shows potential (1) to improve coverage, but "whole-heart" coverage demands high acceleration forcing compromises such as loss of spatial resolution. 2D FPP shows high diagnostic ability with more slices distributed over alternate-RR cycles (2), relaxing acceleration requirements. This work proposes that 3D FPP could interleave two strategies with different 3D parameters in alternate cycles, in sum approaching the full set of desired FPP properties. This work also aims to improve specificity against artefacts by imaging the same myocardium with two different interleaved 3D scans (distinct from reformatting a single 3D FPP scan). Similar confirmation strategies are often used in CMR, such as repetition with swapped phase-encode direction in late-enhancement imaging.

## Methods

**Figure 1:**
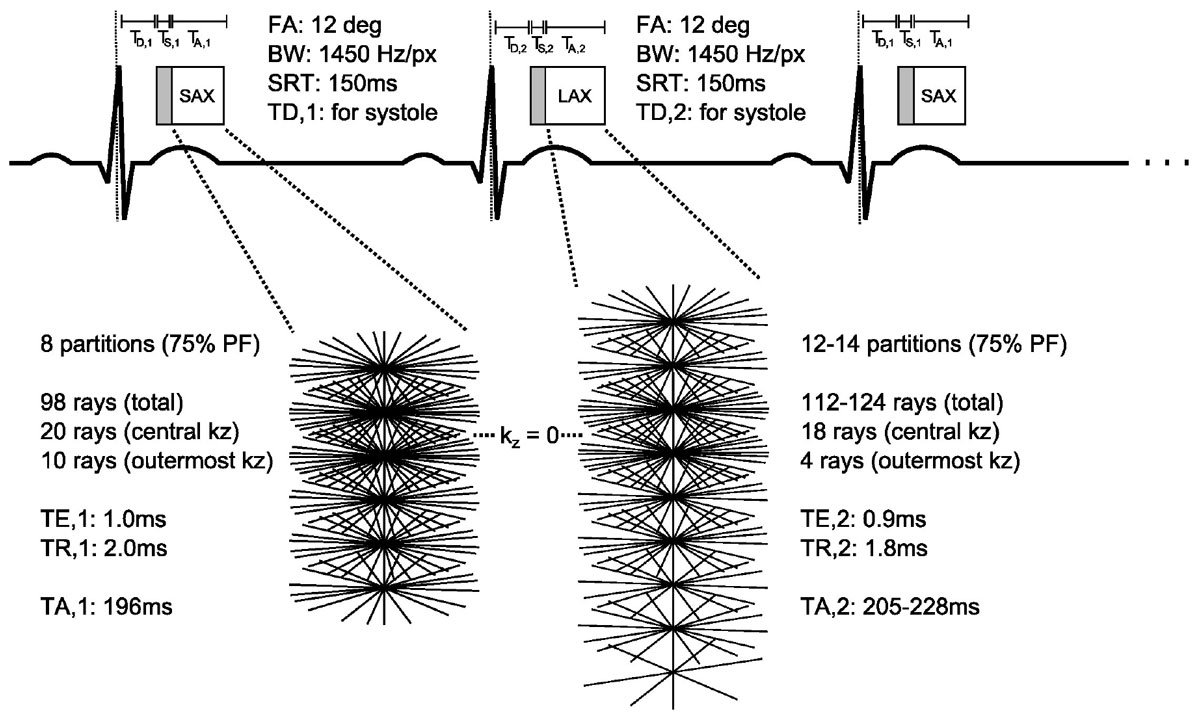
**The alternate-RR 3D FPP protocol**. High in-plane resolution SAX data is acquired on even numbered cardiac cycles, with fewer acquired kz partitions along the LV. On odd cycles, the LAX orientation and systolic acquisition combine to minimise the FOV required by partition encoding, enabling higher through-plane and lower in-plane resolution towards isotropic. Rays per partition gradually reduce for further acceleration. Zero-filled partial Fourier is applied with additional zero padding in-plane (for SAX) and through-plane (for LAX) during reconstruction.

Spoiled gradient-echo 3D radial ‘stack-of-stars' imaging (3) was developed for independent FOV, resolution and position on alternate cardiac cycles. After extensive volunteer optimisation, rest perfusion was acquired in 10 patients with SAX images of higher in-plane resolution acquired on odd cardiac cycles and lower but more isotropic resolution LAX images on even cycles (Figure 1). The sequence was highly optimised for speed, e.g. through use of high undersampling, custom-tailored RF pulses and asymmetric readouts (75%). The settings of the alternate SAX and LAX cycles are shown in Figure 1. The SAX cycles obtain 6-8 wrap-free slices reconstructed as 1.1 × 1.1 × 10.0 mm voxels. The LAX cycles obtain 20-24 usable reconstructed slices at 3.1 × 3.1 × 3.1 mm. A total variation temporally constrained algorithm was used for reconstruction (4) with temporal weighting = 0.7 and 50 iterations.

## Results

**Figure 2:**
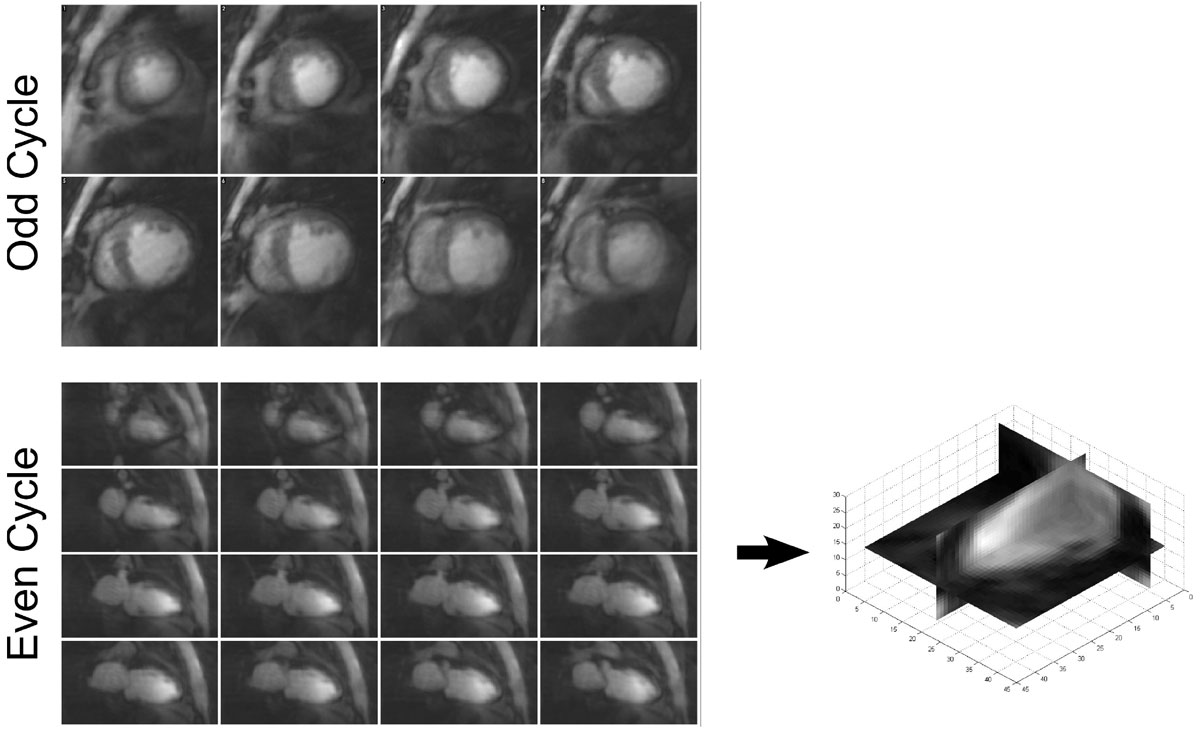
**Example images from a single patient with DCM, showing slices of the higher in-plane resolution SAX frame (top) and 16 slices of an isotropic lower-resolution LAX frame (bottom) during the first pass**. Example reformatted slices from the LAX acquisition are shown on the right to demonstrate the 3D isotropic nature of the reconstructed images. The thin myocardial wall, even during systole, demonstrates the requirement for high resolution imaging in this protocol.

All patients were successfully imaged by this method at higher in-plane and through-plane resolutions than previously achieved by 3D FPP for the SAX and LAX acquisitions respectively (e.g. Figure 2), in realistic acquisition times. The SAX images produce higher quality images, similar to 2D FPP but with contiguous coverage and showing all slices at the same cardiac phase. The LAX images give the reader a second viewpoint with altered motion and resolution characteristics aiding artefact judgement, although they can also be reformatted into any plane. Image quality in the LAX is reduced, but moves towards isotropic reconstructed resolution images while potentially adding information to the more conventional SAX acquisition.

## Conclusions

The feasibility of alternate-RR separate 3D acquisitions during a single FPP is presented, capable of acquiring datasets that stretch current limits of both in-plane and through-plane resolutions while delivering two independently optimised acquisitions of the same first-pass for potentially improving clinical confidence.

## References

[1] Fair (2015). JCMR.

[2] Bertschinger (2001). JMRI.

[3] Chen (2012). Med Phys.

[4] Adluru (2007). MRM.

